# Thrombotic Complications in Patients With Coronavirus Disease 2019 (COVID-19): Two Contrasting Clinical Presentations Viewed Through the Concept of Thromboinflammation

**DOI:** 10.7759/cureus.117525

**Published:** 2026-10-06

**Authors:** Can Colakoglu

**Affiliations:** 1 Department of Intensive Care Medicine, Health Sciences University (HSU) Dr. Suat Seren Chest Diseases and Chest Surgery Training and Research Hospital, Izmir, TUR

**Keywords:** arterial thrombosis, catheter embolectomy, covid-19, thromboinflammation, thrombotic thrombocytopenic purpura

## Abstract

Thrombotic complications are well-recognized extrapulmonary manifestations of coronavirus disease 2019 (COVID-19). Although initially attributed to virus-specific endothelial injury, increasing evidence suggests that these events are better explained by thromboinflammatory mechanisms involving immunothrombosis. We present two contrasting thrombotic complications observed during acute severe acute respiratory syndrome coronavirus 2 (SARS-CoV-2) infection and discuss them within the framework of thromboinflammation and host susceptibility. The first patient was a previously healthy 35-year-old postpartum woman who developed acquired thrombotic thrombocytopenic purpura (TTP) characterized by microangiopathic hemolytic anemia, severe thrombocytopenia, acute kidney injury, neurological deterioration, reduced ADAMTS13 activity, and a favorable response to therapeutic plasma exchange. Despite hematological recovery, distal ischemic injury resulted in digital amputation. The second patient was a 61-year-old man with a heavy smoking history and a radiologically suspicious pulmonary malignancy who developed acute right femoral artery thrombosis during severe COVID-19 pneumonia, requiring emergency thromboembolectomy. Although revascularization was successful, distal tissue necrosis developed, whereas ischemic manifestations in the remaining extremities resolved with combined medical therapy. Both cases demonstrated distinct thrombotic phenotypes despite a common inflammatory trigger. These contrasting cases support the concept that severe systemic inflammation may produce different thrombotic manifestations according to underlying host susceptibility rather than reflecting a unique SARS-CoV-2-specific vasculopathy. The COVID-19 pandemic provided an unprecedented opportunity to observe diverse thromboinflammatory phenotypes, contributing to a broader understanding of thromboinflammation beyond COVID-19 itself.

## Introduction

Coronavirus disease 2019 (COVID-19) was initially recognized as a predominantly respiratory illness characterized by pneumonia and hypoxemic respiratory failure [1]. As clinical experience accumulated, however, it became evident that thrombotic complications represented an important contributor to morbidity and mortality among affected patients [2,3]. Venous thromboembolism, arterial thrombosis, thrombotic microangiopathy-like syndromes, and disseminated intravascular coagulation-like presentations have all been reported across a broad spectrum of disease severity [2-4]. The unprecedented number of critically ill patients during the pandemic provided a unique opportunity to observe these uncommon vascular manifestations more frequently than in routine clinical practice, stimulating extensive investigation into their underlying mechanisms. 

Early reports proposed that severe acute respiratory syndrome coronavirus 2 (SARS-CoV-2) might directly infect endothelial cells through angiotensin-converting enzyme 2 (ACE2) receptors, resulting in endothelial injury and diffuse endotheliitis [5]. Histopathological studies demonstrating endothelial disruption, microvascular thrombosis, and angiogenesis further supported the concept of COVID-19 as a unique vascular disease [6,7]. Consequently, the terms "COVID-19-associated vasculopathy" and "COVID-19-associated coagulopathy" entered the scientific literature to describe these phenomena [5-7]. 

However, subsequent evidence suggested that the pathophysiology of thrombosis in COVID-19 is more complex than direct virus-induced vasculopathy alone. Activation of innate immune pathways, excessive cytokine release, complement activation, platelet activation, neutrophil extracellular trap (NET) formation, and tissue factor expression collectively contribute to a thromboinflammatory state known as immunothrombosis [8-10]. Although these mechanisms promote endothelial dysfunction and coagulation activation, they are not unique to SARS-CoV-2 infection and have also been described in other severe infections and hyperinflammatory conditions [8-10]. 

Importantly, thrombotic complications during COVID-19 are remarkably heterogeneous. While some patients develop predominantly microvascular manifestations resembling thrombotic microangiopathy, others experience large-vessel arterial thrombosis despite exposure to a similar inflammatory stimulus. These observations suggest that the clinical expression of thrombosis may reflect not only the intensity of systemic inflammation but also the underlying susceptibility of the host [4]. 

In this report, we present two contrasting cases observed during the COVID-19 pandemic: one patient with thrombotic microangiopathy requiring therapeutic plasma exchange and another with femoral artery thrombosis requiring emergency surgical thromboembolectomy. Rather than interpreting these cases as evidence of a unique COVID-19-specific vasculopathy, we discuss them within the current concept of thromboinflammation, in which a common immunothrombotic response may give rise to distinct clinical presentations according to individual host susceptibility. By integrating these cases with the current literature, we aim to illustrate how the COVID-19 pandemic provided a unique opportunity to advance our clinical understanding of thromboinflammatory vascular disease beyond SARS-CoV-2 infection itself.

Case 1 was presented at the 19th Annual Congress of the Society of Turkish Intensivists and the 11th Euro-Asian Critical Care Meeting on November 9-12, 2022 (poster PS-08; J Crit Intensive Care. 2022;13[Suppl 1]). Case 2 was presented at the 18th Annual Congress of the Turkish Society of Medical and Surgical Intensive Care Medicine and the 10th Euro-Asian Critical Care E-Meeting on December 6-9, 2021 (poster PS-02; J Crit Intensive Care. 2021;12[Suppl 1]).

## Case presentation

Case 1 

A 35-year-old previously healthy woman, three months postpartum, presented to the internal medicine department with complaints of fatigue and malaise. Initial laboratory investigations revealed elevated liver enzymes and acute kidney injury, prompting hospital admission for further evaluation. During the first day of hospitalization, she developed progressive dyspnea, and a nasopharyngeal reverse transcriptase-polymerase chain reaction (RT-PCR) test for SARS-CoV-2 returned positive. She was subsequently transferred to the intensive care unit (ICU) because of severe SARS-CoV-2 infection with multi-organ involvement. 

On ICU admission, the patient was conscious but lethargic, with a Glasgow Coma Scale (GCS) score of 12 [11]. Her respiratory rate was 24-28 breaths/min, her blood pressure was 150/90 mmHg, and her heart rate was 96 beats/min in sinus rhythm. Physical examination revealed cyanosis and necrotic changes involving the distal portions of all four extremities (Figure 1). Pulse oximetry failed to detect oxygen saturation because of severe peripheral hypoperfusion. Analgesia and supplemental oxygen therapy (4 L/min via nasal cannula) were initiated. Intravenous unfractionated heparin (5,000 IU) was administered initially, followed by enoxaparin at a dose of 40 mg twice daily. Arterial blood gas analysis demonstrated preserved oxygenation, with a PaO₂/FiO₂ ratio exceeding 200. Contrast-enhanced thoracic and peripheral computed tomography angiography revealed no evidence of large-vessel arterial occlusion.

**Figure 1:**
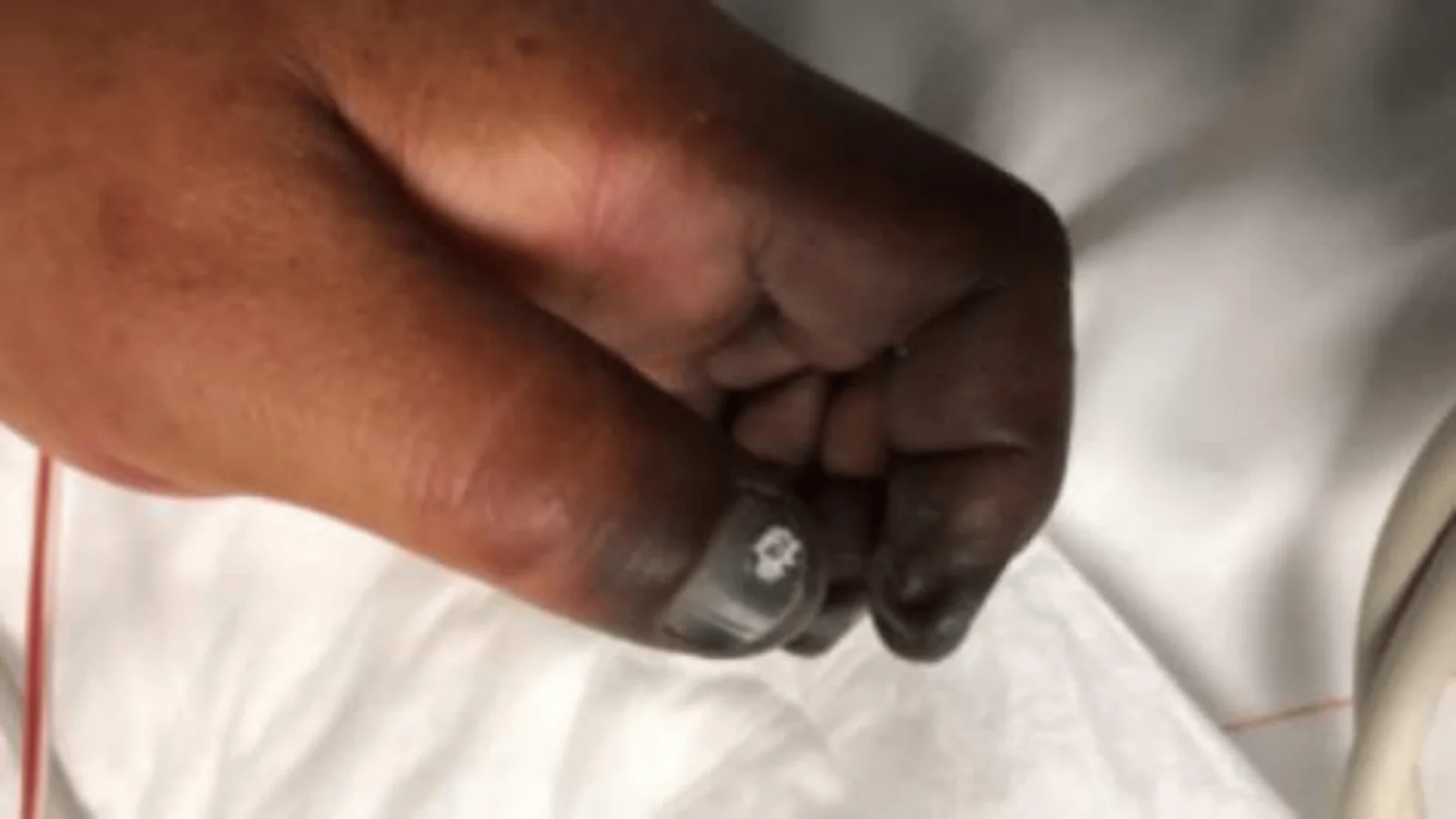
Distal ischemic changes of the upper extremity at intensive care unit admission. Progressive cyanosis and dry gangrene involving the fingertips developed despite the absence of angiographically detectable large-vessel occlusion, suggesting a predominantly microvascular thrombotic process

The constellation of microangiopathic hemolytic anemia, severe thrombocytopenia, acute kidney injury, and schistocytosis raised suspicion for thrombotic microangiopathy. Differential diagnoses included acquired thrombotic thrombocytopenic purpura (TTP), atypical hemolytic uremic syndrome (aHUS), postpartum-associated thrombotic microangiopathy, disseminated intravascular coagulation, catastrophic antiphospholipid syndrome, and systemic vasculitis. Given the absence of marked coagulation abnormalities and the urgency of treatment, acquired TTP was considered the most likely diagnosis. ADAMTS13 activity testing was requested; however, therapeutic plasma exchange was initiated without awaiting the results because delaying treatment could have resulted in a fatal outcome. Diagnostic investigations supporting thrombotic microangiopathy are summarized in Table 1.

**Table 1:** Diagnostic evaluation supporting the diagnosis of thrombotic thrombocytopenic purpura

Parameter	Result	Unit	Reference range
Hemoglobin	7.1	g/dL	12.0-16.0
Platelet count	27	×10⁹/L	150-400
Lactate dehydrogenase	1081	U/L	120-246
Serum creatinine	3.3	mg/dL	0.5-1.1
Blood urea nitrogen	116	mg/dL	7-20
Total bilirubin	0.9	mg/dL	0.3-1.2
Direct bilirubin	0.5	mg/dL	0.0-0.3
International normalized ratio	1.17	-	0.8-1.2
Activated partial thromboplastin time	35	s	25-35
Peripheral blood smear	Schistocytes	-	-
Complement C3	Within normal limits	-	-
Complement C4	Within normal limits	-	-
Antinuclear antibody	Negative	-	-
Antineutrophil cytoplasmic antibody	Negative	-	-
ADAMTS13 activity	<20	%	>60

Following hematology consultation, daily therapeutic plasma exchange was initiated and continued for five consecutive days. High-dose dexamethasone (40 mg/day) was administered concomitantly as adjunctive immunosuppressive therapy for suspected immune-mediated TTP. Platelet and red blood cell transfusions were avoided unless deemed clinically necessary. Intravenous iloprost infusion was started early to improve peripheral microcirculatory perfusion at a dose of 40 μg/day. 

On the second day of ICU follow-up, the patient's level of consciousness deteriorated, and she became responsive only to painful stimuli. Endotracheal intubation was performed for airway protection, and invasive mechanical ventilation was initiated. During treatment, renal function progressively improved, liver enzyme abnormalities resolved, and hematological parameters normalized. ADAMTS13 activity was subsequently found to be reduced, further supporting the diagnosis of TTP. The patient was successfully liberated from mechanical ventilation after six days of ventilatory support and was extubated on ICU day 7. She was discharged from the ICU on day 10 and subsequently transferred to the general ward. Following a total hospital stay of 19 days, she was discharged from the hospital. 

Despite hematological recovery and resolution of organ dysfunction, distal ischemic injury progressed to irreversible necrosis of the affected extremities. The patient ultimately required distal amputation because of persistent ischemic tissue loss. 

The overall clinical presentation, reduced ADAMTS13 activity, and favorable response to plasma exchange and corticosteroid therapy supported the diagnosis of acquired TTP. The postpartum period likely represented an important host susceptibility factor, whereas concurrent SARS-CoV-2 infection may have served as a potent inflammatory trigger rather than the sole etiologic factor in the development of TTP.

Case 2 

A 61-year-old man with a 50 pack-year smoking history and no known chronic medical conditions presented to the emergency department with a five-day history of cough, fever, and progressively worsening dyspnea. On admission, his respiratory rate was 28 breaths/min, his peripheral oxygen saturation was 84% on room air, his blood pressure was 130/80 mmHg, his heart rate was 108 beats/min, and his body temperature was 38.3°C. Bilateral inspiratory crackles were auscultated predominantly over the lower lung fields. 

Chest radiography demonstrated bilateral patchy air-space infiltrates involving the middle and lower lung zones. A well-defined left upper lung opacity suspicious for an underlying pulmonary mass was also evident. Thoracic computed tomography confirmed bilateral peripheral and basal predominant ground-glass opacities consistent with COVID-19 pneumonia and further characterized the left upper lobe lesion as a lobulated spiculated solid mass highly suspicious for primary lung malignancy (Figure 2). Serologic testing was positive for SARS-CoV-2 IgM and negative for IgG, supporting a diagnosis of acute SARS-CoV-2 infection.

**Figure 2:**
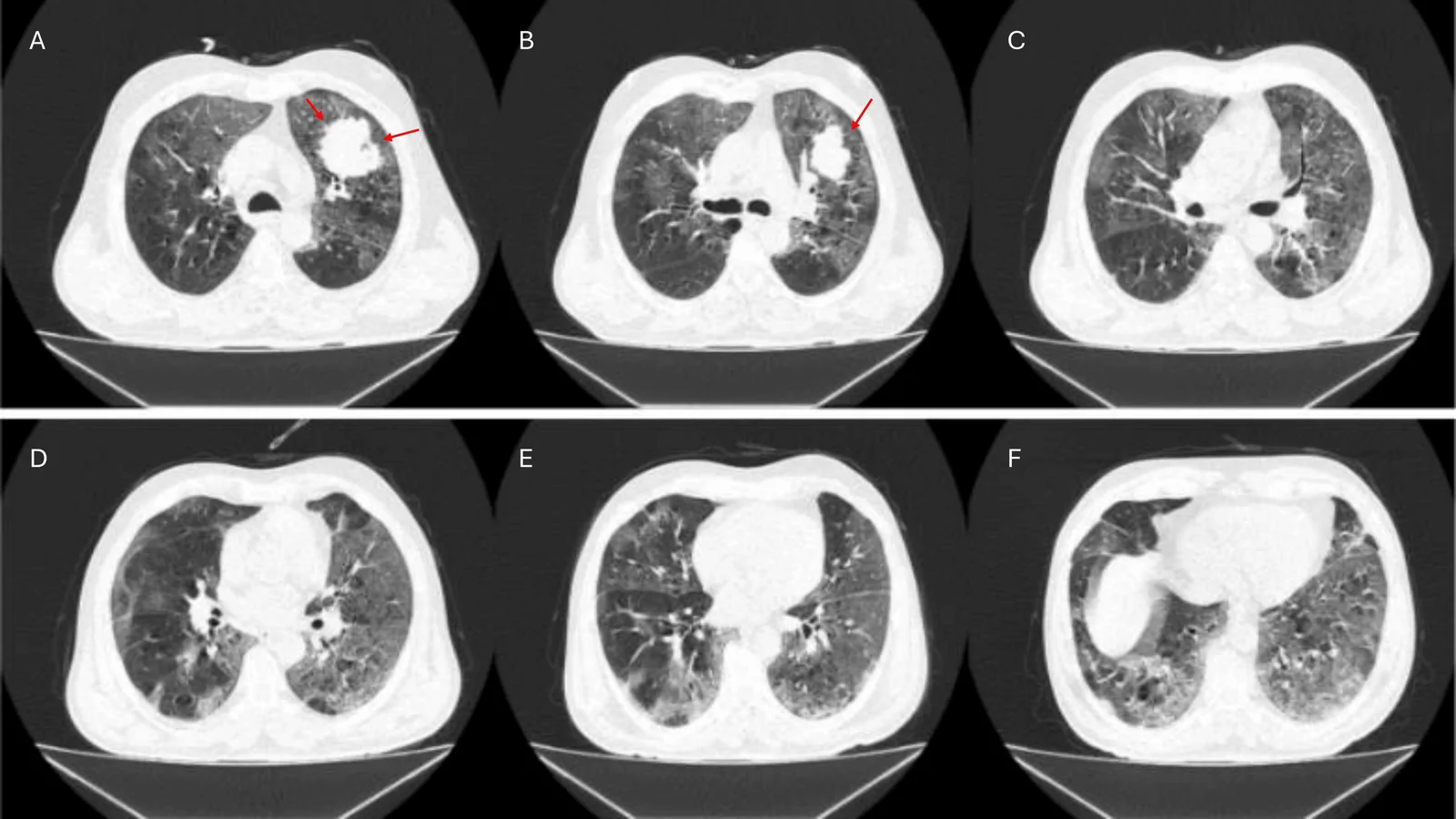
Thoracic computed tomography findings. (A-B) Axial lung-window images demonstrate a lobulated, spiculated solid mass in the left upper lobe (arrows), highly suspicious for primary lung malignancy. (C-F) Serial axial images demonstrate bilateral, predominantly peripheral, and basal ground-glass opacities, consistent with COVID-19 pneumonia

The patient was admitted to the ICU because of acute hypoxemic respiratory failure requiring non-invasive mechanical ventilation. Initial treatment consisted of favipiravir, enoxaparin (60 mg twice daily), systemic corticosteroids, nebulized budesonide, supplemental oxygen, and supportive care. 

On ICU day 3, the patient developed pain, numbness, and coldness in the right lower extremity. Simultaneously, impaired peripheral perfusion and coldness were observed in the left lower extremity and in the distal portions of both hands. Acetylsalicylic acid (100 mg/day), clopidogrel (75 mg/day), and intravenous iloprost infusion (40 μg/day) were added because of progressive ischemic findings. Laboratory findings obtained at the onset of acute arterial thrombosis are summarized in Table 2.

**Table 2:** Laboratory findings at the onset of acute arterial thrombosis

Parameter	Result	Unit	Reference range
White blood cell count	11.6	×10³/µL	4.0-10.0
Hemoglobin	13.9	g/dL	13.0-17.0
Platelet count	296	×10³/µL	150-400
Absolute lymphocyte count	0.6	×10³/µL	1.0-4.0
Absolute neutrophil count	10.2	×10³/µL	1.5-7.5
Creatinine	0.40	mg/dL	0.7-1.3
Urea	25.6	mg/dL	17-43
Aspartate aminotransferase	81	U/L	12-38
Alanine aminotransferase	41	U/L	10-40
Lactate dehydrogenase	934	U/L	120-246
Total bilirubin	0.66	mg/dL	0.3-1.2
Albumin	2.22	g/dL	3.5-5.5
Sodium	132	mmol/L	136-145
Potassium	4.82	mmol/L	3.5-5.1
C-reactive protein	13.85	mg/dL	<0.5
D-dimer	2777	ng/mL FEU	<500
Neutrophil-to-lymphocyte ratio	17.0	-	-
Procalcitonin	0.49	ng/mL	<0.5

Computed tomography angiography demonstrated complete absence of contrast enhancement distal to the right femoral artery, consistent with acute arterial occlusion (Figure 3). Cardiovascular surgery consultation was obtained, and the patient underwent catheter-assisted femoral thromboembolectomy. Following revascularization, aggressive hydration, anticoagulation, and close biochemical monitoring were initiated because of concern for reperfusion injury.

**Figure 3:**
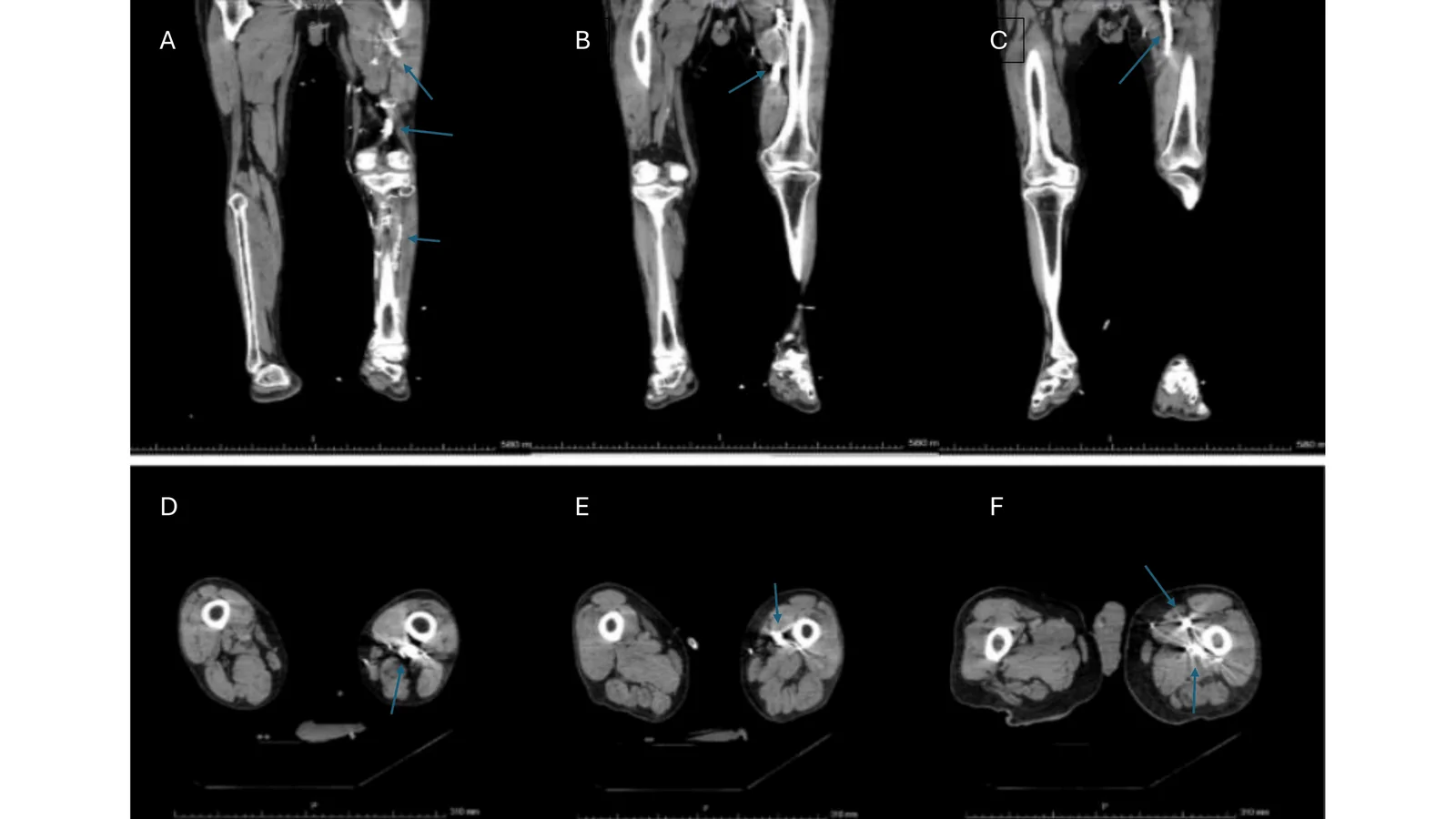
Computed tomography angiography of the lower extremities. (A-C) Coronal reformatted images demonstrate the abrupt interruption of contrast opacification at the right common femoral artery with absent distal arterial filling. (D-F) Axial images at sequential levels confirm the non-opacification of the right femoral arterial system compared with the patent contralateral vessels. Arrows indicate the thrombotic occlusion and the involved arterial segments

Following successful revascularization, the patient developed reperfusion-related metabolic complications requiring temporary continuous renal replacement therapy, from which he subsequently recovered. Despite successful restoration of pulsatile flow in the posterior tibial artery, progressive distal tissue necrosis developed in the right foot. In contrast, the ischemic manifestations involving the left lower extremity and both upper extremities resolved completely with combined anticoagulant, antiplatelet, vasodilator, and supportive therapy. 

The patient's respiratory status gradually improved during ICU follow-up. Renal function recovered sufficiently to allow the discontinuation of renal replacement therapy, and oxygen requirements progressively decreased. At ICU discharge, persistent distal necrosis of the right foot remained, and reconstructive surgical follow-up was planned. 

The final diagnosis was acute limb ischemia secondary to right femoral artery thrombosis occurring during acute SARS-CoV-2 infection. Although histopathological confirmation was unavailable, the coexistence of a suspicious pulmonary malignancy, extensive smoking history, and simultaneous macrovascular and peripheral ischemic manifestations suggests that severe systemic inflammation may have precipitated thrombosis in a susceptible host rather than representing a unique virus-specific vasculopathy.

## Discussion

Thrombotic complications rapidly emerged as one of the most important extrapulmonary manifestations of COVID-19 during the pandemic. Early reports documented unexpectedly high rates of venous thromboembolism, arterial thrombosis, disseminated intravascular coagulation-like syndromes, and thrombotic microangiopathy, leading to the hypothesis that SARS-CoV-2 might represent a unique vascular pathogen [2-4]. Initial studies emphasized direct viral endothelial injury and endotheliitis; however, subsequent experimental and clinical evidence has shifted the focus toward a broader thromboinflammatory paradigm [8-10]. Endothelial activation, platelet activation, complement dysregulation, NET formation, cytokine-mediated inflammation, and activation of the coagulation cascade are now recognized as interconnected components of immunothrombosis rather than isolated pathological events [8-10]. 

The two patients presented here illustrate the remarkable clinical heterogeneity of thrombotic complications observed during COVID-19. Although both patients were exposed to the same infectious stimulus, one developed immune-mediated thrombotic microangiopathy requiring therapeutic plasma exchange, whereas the other experienced acute femoral artery thrombosis requiring emergency thromboembolectomy. Rather than representing two unrelated diseases, these contrasting presentations support the concept that thromboinflammation may constitute a common biological process capable of producing distinct clinical phenotypes according to underlying host susceptibility [4,12]. 

An important implication of these observations is that the clinical expression of thromboinflammation appears to depend not only on the intensity of systemic inflammation but also on underlying host susceptibility [4,12]. In the first patient, the postpartum period may have created an immunological milieu favoring the development of acquired TTP. In the second patient, extensive smoking history together with a radiologically suspicious pulmonary malignancy may have represented a pre-existing prothrombotic substrate upon which acute systemic inflammation precipitated large-vessel arterial thrombosis. From this perspective, SARS-CoV-2 may have acted primarily as a potent inflammatory trigger rather than as the sole etiological factor responsible for either vascular complication [4,12]. 

This interpretation is supported by observations beyond COVID-19. Similar thrombotic syndromes have long been described in association with influenza, bacterial sepsis, HIV infection, malignancy, autoimmune disorders, and other hyperinflammatory conditions [13]. The biological pathways implicated in these disorders, including endothelial dysfunction, complement activation, platelet activation, and NET-mediated immunothrombosis, are not unique to SARS-CoV-2. Consequently, the central question may be less whether COVID-19 causes a distinct virus-specific vasculopathy and more why the pandemic allowed clinicians to observe such an extraordinary diversity of thrombotic syndromes. 

The unprecedented scale of the COVID-19 pandemic provides a plausible explanation. Millions of individuals worldwide were simultaneously exposed to a common inflammatory stimulus, allowing relatively uncommon thrombotic disorders to be recognized with unprecedented frequency. In this sense, the pandemic functioned as a natural clinical model through which the broader biology of thromboinflammation became more apparent. Rather than discovering entirely new vascular diseases, clinicians gained a unique opportunity to observe familiar thromboinflammatory mechanisms expressed across diverse vascular beds and clinical settings. 

Our cases also highlight the close relationship between microvascular and macrovascular thrombosis. In the first patient, distal tissue necrosis developed despite the absence of angiographically detectable large-vessel occlusion, supporting a predominantly microvascular mechanism. Conversely, the second patient demonstrated angiographically confirmed femoral artery thrombosis while simultaneously exhibiting reversible ischemic changes in other extremities, suggesting concurrent microcirculatory dysfunction. These findings support the concept that microvascular and macrovascular thrombosis should not necessarily be viewed as independent processes but rather as different clinical expressions of overlapping thromboinflammatory pathways [4,9,10,13]. 

Both patients received adjunctive iloprost therapy because of severe peripheral ischemic manifestations. Although no conclusions regarding therapeutic efficacy can be drawn from two cases, the favorable evolution of peripheral perfusion in selected vascular territories suggests that prostacyclin analogues deserve further evaluation as adjunctive therapies in thromboinflammatory ischemic syndromes [14,15]. Prospective controlled studies will be required to determine whether these agents provide additional benefit beyond conventional antithrombotic treatment. 

Several limitations should be acknowledged. As with all case reports, causal relationships cannot be established. Both patients had potential susceptibility factors independent of SARS-CoV-2 infection, including the postpartum state in the first patient and heavy smoking with suspected pulmonary malignancy in the second. Consequently, these observations should be regarded as hypothesis-generating rather than definitive evidence that SARS-CoV-2 directly caused the observed vascular complications.

## Conclusions

The COVID-19 pandemic provided an unprecedented opportunity to observe diverse thrombotic manifestations associated with severe systemic inflammation. Our two contrasting cases suggest that similar inflammatory stimuli may give rise to distinct vascular phenotypes according to underlying host susceptibility. Rather than supporting the concept of a unique SARS-CoV-2-specific vasculopathy, these observations reinforce the view that thromboinflammation represents a common biological pathway linking infection, inflammation, and thrombosis. Understanding this host-dependent variability may prove valuable not only for COVID-19 but also for thrombotic complications arising in other infectious and hyperinflammatory diseases.
